# Assessing donor kidney function: the role of CIRBP in predicting delayed graft function post-transplant

**DOI:** 10.3389/fimmu.2024.1518279

**Published:** 2025-01-17

**Authors:** Qianghua Leng, Maolin Ma, Zuofu Tang, Weichen Jiang, Fei Han, Zhengyu Huang

**Affiliations:** Organ Transplantation Research Institution, Division of Kidney Transplantation, Department of Surgery, The Third Affiliated Hospital of Sun Yat-sen University, Guangzhou, Guangdong, China

**Keywords:** kidney transplantation, cold-inducible RNA binding protein, delayed graft function, graft survival, acute kidney injury

## Abstract

**Introduction:**

Delayed graft function (DGF) shortens the survival time of transplanted kidneys and increases the risk of rejection. Current methods are inadequate in predicting DGF. More precise tools are required to assess kidney suitability for transplantation. Cold-inducible RNA-binding protein (CIRBP) expression has been linked to acute kidney injury, suggesting its potential as a new biomarker for transplanted kidney function.

**Methods:**

We included deceased donors and recipients who had undergone successful kidney transplantation between 2016 and 2019. Recipients and their paired donors are assigned to either the DGF or immediate graft function (IGF) group, based on the recipient’s recovery of graft renal function. Donor plasma CIRBP levels were measured using an enzyme-linked immunosorbent assay kit to assess their relationships with DGF.

**Results:**

Donor plasma CIRBP concentrations in the DGF group were approximately twice as high as those in the IGF group (6.82 vs. 3.44; P<0.001). DGF occurred in all cases where CIRBP concentrations exceeded 7.92 ng/mL. Furthermore, univariate and multivariate analyses (odds ratio [OR]=1.660; P<0.001) confirmed that donor plasma CIRBP level was an independent risk factor for DGF. Additionally, higher CIRBP levels were associated with increased plasma creatinine at 6 months (R²=0.08; P<0.001), and survival analysis showed shorter kidney survival in recipients with DGF (P=0.002).

**Conclusions:**

This study demonstrated that donor plasma CIRBP levels can effectively predict the occurrence of DGF. CIRBP is a potential novel biomarker for evaluating transplanted kidney function.

**Clinical trial registration:**

https://clinicaltrials.gov, identifier NCT06641622.

## 1 Introduction

Research has shown that delayed graft function (DGF) significantly reduces the overall survival rate of transplanted kidneys, increases the risk of rejection, prolongs hospital stay, and leads to higher healthcare costs (1). The adoption of extended-criteria donor (ECD) kidneys has expanded in recent years to address donor kidney shortages. However, this expansion has caused increased DGF incidence and graft loss (2). Thus, more accurate methods for assessing graft function and predicting DGF are essential to optimize the safe use of ECD donor kidneys. Current biomarkers for assessing kidney function are difficult to implement in clinical settings, and existing scoring systems lack sufficient validation (3).

Serum creatinine is a kidney-specific metabolite most widely used as a marker for evaluating renal function (4). Its levels at various time points are also significant in predicting early outcomes in transplanted kidneys (5). However, serum creatinine levels are influenced by variables like age, sex, and muscle mass, and it does not accurately reflect tubular damage, limiting its clinical utility (6). Biomarkers such as exosomal miRNAs (7), transcriptomics UBD mRNA (8), and Klotho (9) are associated with DGF; however, they have been studied in small sample sizes, and their detection methods are complex, hindering the establishment of a unified clinical diagnostic standard. The kidney donor profile index (KDPI) is a widely used indicator for predicting the long-term survival of transplanted kidneys across various kidney transplant centers. However, the KDPI lacks comprehensive patient data, limiting its accuracy and clinical applications (10). Several existing scoring systems exhibit limited predictive ability for transplant kidney outcomes (11). Although incorporating pathological findings of the transplanted kidney into these scoring systems improves the accuracy of DGF prediction, it remains time-consuming and does not support timely decision-making (12).

Cold-inducible RNA-binding protein (CIRBP), has been classified as a damage-associated molecular pattern (DAMP), belongs to the heterologous ribonucleoprotein subgroup within the RNA-binding protein family (13). Also known as heterologous ribonucleoprotein A18, CIRBP responds to various stress conditions by modulating mRNA stability (14). Under normal conditions, CIRBP is expressed at low levels in tissues, resulting in low serum concentrations. However, when tissues experience damage from factors such as low temperature, oxidative stress, or ultraviolet irradiation, CIRBP expression increases significantly in the cytoplasm and is released extracellularly via the lysosomal and exosomal pathways (15). Serum CIRBP levels can increase within 6 h of tissue injury (16). During acute kidney injury (AKI), epithelial cells express and release large amounts of CIRBP, leading to cellular dysfunction by promoting the production of reactive oxygen species (ROS) and the cleavage of mitochondrial double-stranded DNA (17), inducing the release of inflammatory factors and apoptosis and accelerating AKI progression (18). Extracellular CIRBP worsens renal injury by binding to TREM-1 during ischemia-reperfusion injury (IRI) (19). Clinical studies have demonstrated that postoperative cardiac surgery patients with elevated serum CIRBP levels are more likely to develop AKI, with those exhibiting above-average concentrations experiencing more severe injury (20, 21). Therefore, donor serum CIRBP levels reflect kidney injury before transplantation and can serve as a possible indicator for predicting post-transplant graft outcomes. Additionally, CIRBP is more clinically applicable than other biomarkers due to the ease of obtaining donor specimens and the convenience of the assay method.

The purpose of this study was to investigate the relationship between serum CIRBP levels and kidney graft function following transplantation, with the hypothesis that elevated CIRBP levels are indicative of severe donor kidney injury and reduced post-transplant graft function.

## 2 Materials and methods

### 2.1 Study design

This retrospective study involved deceased donors and recipients who had undergone successful kidney transplantation in our transplant center between 2016 and 2019. No organs were sourced from executed prisoners. Donors were excluded if they fulfilled any of the following criteria: (a) lacked a blood specimen, (b) had contaminated blood specimens affecting CIRBP levels, (c) were recipients who underwent combined multi-organ transplants, (d) were participating in other clinical trials, (e) had a history of organ transplantation, or (f) had other conditions deemed unsuitable for enrollment by the investigator. Blood specimens were collected from donors by Organ Procurement Organizations and used in various studies with the donors’ consent. All recipients were followed-up for a long period post-transplantation.

This study was conducted in accordance with the ethical standards outlined in the Declaration of Helsinki (22) and approved by the Medical Ethics Committee of The Third Affiliated Hospital of Sun Yat-sen University. Organs were allocated in a fair and transparent manner using the Chinese Organ Transplant Response System (23). The donor’s family voluntarily determined the type of donation, and the recipients underwent follow-up after transplantation. All participants provided informed consent before being included in the study, and their confidentiality was ensured throughout the research process. Data were anonymized to protect personal identities, and no patient-identifiable information was used in any part of the study.

Donor data were obtained from the Chinese Computerized System for Human Organ Distribution and Sharing, completed by members of organ transplant organizations. Donor information was obtained from clinical electronic medical records. Recipient data were derived from electronic clinical records alone. Recipients were categorized into the DGF or immediate graft function (IGF) group according to their early renal function status. Donors were similarly classified into DGF and IGF groups, depending on the early renal function recovery of their paired recipients. DGF was defined as a reduction in blood creatinine concentration of less than 10% for three consecutive days within 1 week postoperatively, or if serum creatinine (SCr) did not decrease to 400 μmol/L within 1 week post-surgery (24, 25). IGF was defined as the fast postoperative recovery of renal function with satisfactory diuresis and no further need for dialysis. Graft loss was defined as end stage renal disease requiring dialysis or retransplantation. ECD donors were defined as those over 60 years-old, or between 50 and 59 years old who met at least two of the following criteria: final serum creatinine exceeding 133 μmol/L, cause of death being cerebrovascular accident, or a history of hypertension (26). We converted human leukocyte antigen (HLA) mismatches into hierarchical profiles to facilitate data analysis: 0 to 1 mismatch as Level 1, 2 to 4 mismatches as Level 2, and 5 to 6 mismatches as Level 3.

### 2.2 Sample collection

Prior to kidney procurement, 2 mL of fresh blood was drawn from the donor in the operating room using a vacuum blood collection needle and placed in a sterile test tube. The blood was left at room temperature for 1 hour and then centrifuged at 4°C, 2,500 rpm for 10 min. The resulting supernatant was separated, while the sediment was discarded. The supernatant was subsequently divided into aliquots, frozen, and stored at -80°C in the hospital. Before testing, the serum samples were thawed on crushed ice and centrifuged again at 1,000 g for 15 min at 4°C.

### 2.3 Measurement of cold inducible RNA binding protein level

Serum CIRBP concentrations were measured using an enzyme-linked immunosorbent assay (ELISA) kit (CSB-EL005440HU). Firstly, the protein standard was diluted per the instructions, and 100 µL of different concentrations of standard and samples (1:10 dilution) were added to a 96-well plate in duplicates, and the plates were incubated at 37°C for 2 h in the dark. Following incubation, the liquid from the wells was discarded, and 100 µL of pre-diluted anti-CIRBP biotin-labeled antibody solution was introduced into each well. After incubating at 37°C for 1 h, each well was washed at least three times with a washing solution before 100 μL of diluted horseradish peroxidase-labeled antibody was added to each well, following the same procedure as that in the previous step. After another 1-hour incubation at 37°C, we washed each well at least five times, to prevent false positive results. Thereafter, we added 90 µL of substrate solution to each well, and add 50 µL of termination solution was added to stop the reaction after a 15-min color development at 37°C. The optical density (OD) at 450 nm was measured within 5 minutes using an enzyme marker. A standard curve model was then generated from the OD values of the standard wells, and the CIRBP concentration of each sample was determined using this model.

### 2.4 Statistical analysis

Age is presented as the mean ± standard deviation (SD). Student’s t-test was applied to compare the ages between the DGF and IGF groups. Body mass index (BMI), donor terminal creatinine, donor serum CIRBP, kidney donor risk index, KDPI, dialysis duration, and post-transplantation renal function values are presented as median [interquartile range], and these variables were compared between the DGF and IGF groups using the Mann-Whitney U test. Frequencies and percentages were used to present categorical variables, such as sex, cause of death, and dialysis modality. Comparisons between two groups were performed using the Chi-square tests when the sample size was ≥40 and Fisher’s exact tests when the sample size was <40. KDPI was calculated using standard methods (27, 28). Factors associated with DGF and graft loss were confirmed using the Spearman correlation analysis.

Each factor potentially associated with the occurrence of DGF after renal transplantation was initially analyzed using univariate logistic regression to statistically determine its influence on DGF. Independence of these factors was further evaluated through stepwise backward multivariate logistic regression, with entry criteria set at 0.05 and removal criteria at 0.1. This approach ensured that only meaningful parameters were retained in the final model. The predictive ability of CIRBP for DGF and graft loss was evaluated through receiver operating characteristics (ROC) curve analyses, with optimal cutoff points derived from Youden’s index, J, which is determined as J=sensitivity + specificity – Eqn. 1 (29). In addition, we applied forward stepwise multivariate linear regression analyses to identify the factors influencing recipient SCr concentrations at 6 months post-kidney transplantation. The Kaplan-Meier analysis was used to evaluate the impact of DGF occurrence and elevated donor plasma CIRBP levels on allograft survival time following kidney transplantation.

All statistical tests were performed using SPSS version 27.0 (SPSS Inc., Chicago, IL, USA), and significance was determined by a P-value of less than 0.05.

## 3 Results

### 3.1 Baseline characteristics

**Figure 1:**
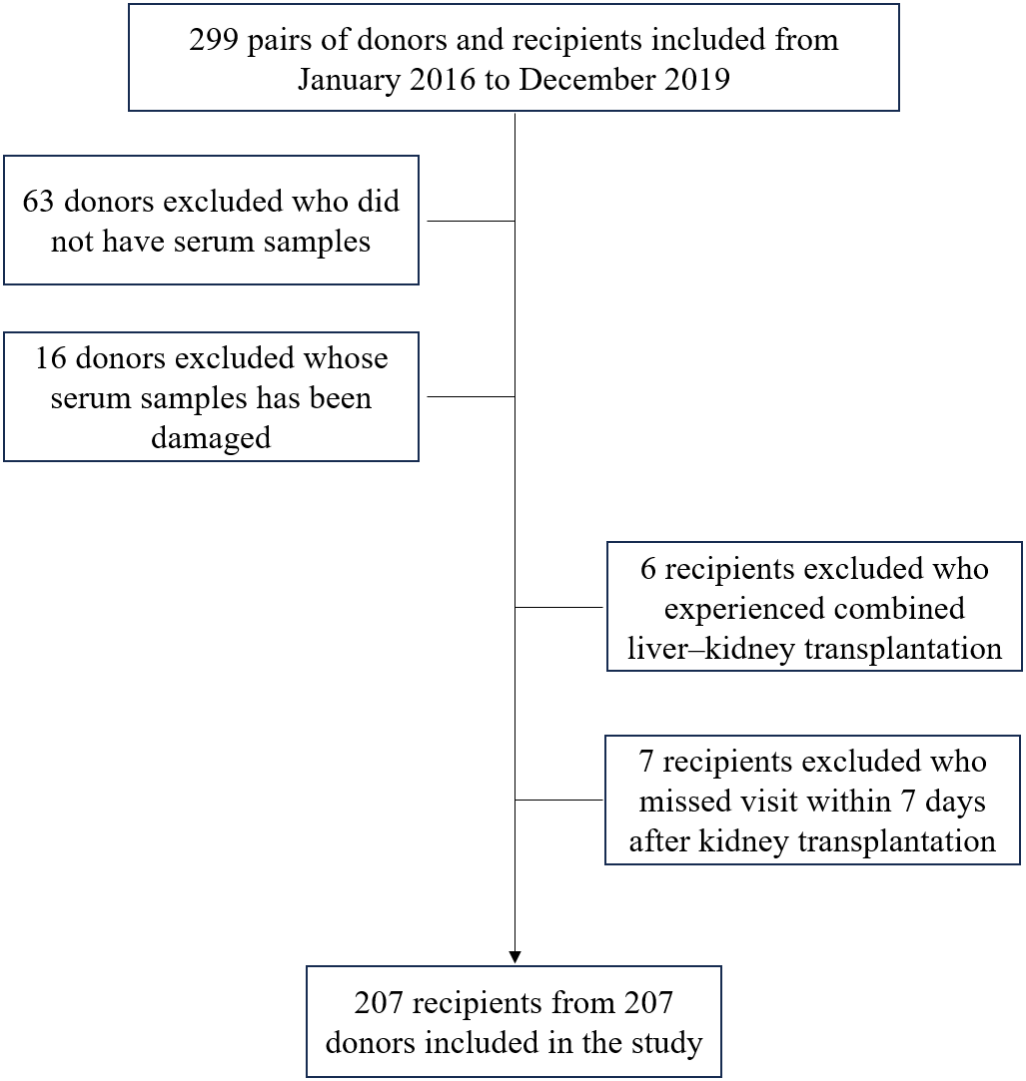
Flow chart of kidney donors and recipients enrolled in the study.

**Figure 1** illustrates the process of assembling the cohort, while **Tables 1** and **2** present the baseline characteristics of the 207 donor-recipient pairs engaged in the study. The cohort was stratified according to allograft function; 51 (25%) recipients developed DGF after kidney transplantation. The DGF and IGF groups were then compared. The mean ± SD donor age was 42.31 ± 14.25 years, and the median [interquartile range] BMI was 23.00 [21.40–25.40] kg/m^2^. Among the donors, 86% were male, 20% had hypertension, 46% died of cerebral hemorrhage, and 17% were extended-criteria donors; however, no factor significantly differed between the groups. As expected, the median plasma CIRBP level in the DGF group was 6.82 ng/mL, significantly higher than that in the IGF group (3.44 ng/mL; P<0.001). Similar results were observed for donor terminal SCr levels (158.00 vs. 109.00; P<0.001). However, we failed to observe the variation in donor KDPI values amidst the DGF and IGF groups (59% vs. 46%; P=0.215), consistent with our prediction that KDPI does not reliably predict early post-transplant renal function. The mean ± SD recipient age was 42.56 ± 10.80 years, with a median [interquartile range] BMI of 21.26 [19.84–22.86] kg/m^2^. Comparing the DGF and IGF groups, we found that male recipients (88% vs. 66%; P=0.002) and those with a higher BMI (22.64 vs. 20.96; P<0.001) had a greater likelihood of developing DGF. Additionally, recipients with DGF had distinctly higher SCr levels at 6 months post-transplant compared to those in the IGF group (171.00 vs. 121.00; P<0.001). No differences were found between the groups in terms of dialysis type, dialysis duration, HLA mismatch level, induction therapy, or immunosuppressant dosage.

**Table 1:** Deceased-donor characteristics, stratified by recipients’ allograft function.

Characteristic	All (n=207)	IGF (n=156)	DGF (n=51)	P
Age (y)	42.31 ± 14.25	42.55 ± 14.21	41.59 ± 14.50	0.676
Male sex	177 (86)	134 (86)	43 (84)	0.780
BMI (kg/m^2^)	23.00 [21.40,25.40]	22.95 [21.50,25.40]	23.00 [21.20,25.40]	0.980
Hypertension	42 (20)	28 (18)	14 (28)	0.143
Cause of death				0.483
Cerebral hemorrhage	96 (46)	69 (44)	27 (52.5)	
Head trauma	93 (45)	72 (46)	21 (42)	
Others	18 (9)	15 (10)	3 (5.5)	
Terminal SCr (μmol/L)	114.00 [77.00,163.00]	109.00 [76.25,144.75]	158.00 [87.00,261.00]	<0.001
KDRI	1.29 [1.11,1.59]	1.26 [1.07,1.59]	1.43 [1.22,1.53]	0.192
KDPI (%)	49 [34,68]	46 [31,68]	59 [42,65]	0.215
Extended criteria donor	35 (17)	25 (16)	10 (20)	0.554
Plasma CIRBP (ng/ml)	4.06 [2.81,6.26]	3.44 [2.45,5.12]	6.82 [4.30,8.89]	<0.001

Continuous variables according to Shapiro test, if P > 0.05, the data are expressed as mean ± SD; otherwise, the data are expressed as median [P25,P75]; categorical variables are described by total numbers and percentages.

Continuous variables were compared using ANOVA or Kruskal–Wallis tests and categorical variables were compared using the χ2 test and Fisher’s exact test.

IGF, immediate graft function; DGF, delayed graft function; BMI, body mass index; KDRI, kidney donor risk index; KDPI, kidney donor profile index; SCr, serum creatinine; CIRBP, cold-inducible RNA binding protein; ANOVA, analysis of variance.

**Table 2:** Recipient characteristics, stratified by recipients’ allograft function.

Characteristic	All (n=207)	IGF (n=156)	DGF (n=51)	P
Age (y)	42.56 ± 10.80	42.42 ± 11.01	43.00 ± 10.24	0.739
Male sex	148 (72)	103 (66)	45 (88)	0.002
BMI (kg/m^2^)	21.26 [19.84,22.86]	20.96 [19.84,22.77]	22.64 [20.96,25.01]	<0.001
Cause of ESRD				0.001
Diabetes	18 (8.5)	10 (6)	8 (15.5)	
GN	66 (32)	60 (38.5)	6 (12)	
PKD	8 (4)	7 (4.5)	1 (2)	
Others	115 (55.5)	79 (51)	36 (70.5)	
Mode of dialysis				0.145
HD	153 (74)	110 (71)	43 (84)	
PD	31 (15)	26 (17)	5 (10)	
Dialysis duration, mo	12.00 [4.00,24.00]	12.00 [4.00,24.00]	12.00 [6.00,26.00]	0.278
Level of HLA mismatches				0.108
Level 1	14 (7)	9 (6)	5 (10)	
Level 2	90 (43)	63 (40)	27 (53)	
Level 3	103 (50)	84 (54)	19 (37)	
Panel reactive antibody				
0%	207	156	51	
1%-10%	0	0	0	
Induction regimen				0.175
ATG	134 (65)	105 (67)	29 (57)	
Basiliximab	73 (35)	51 (33)	22 (43)	
CNI				0.177
Tacrolimus	185 (89)	142 (91)	43 (84)	
Cyclosporin	22 (11)	14 (9)	8 (16)	
Steroid	207	156	51	
Posttransplantation renal function				
SCr, at 1 week, μmol/L	148.00 [105.00,394.00]	128.00 [101.00,168.00]	583.00 [468.00,886.00]	<0.001
SCr, at 6 months, μmol/L	133.00 [106.50,169.50]	121.00 [96.00,151.00]	171.00 [139.00,274.00]	<0.001

Continuous variables according to the Shapiro test, if P > 0.05, the data are expressed as mean ± SD; otherwise data are expressed as median [P25,P75]; categorical variables are described by numbers and percentages (%).

Continuous variables were compared using ANOVA or Kruskal–Wallis tests, categorical variables were compared using the χ2 test and Fisher’s exact test. If the small letters above the data of the 2 groups are different, there was a significant difference between the 2 groups; if they are same, there was no significant difference.

ESRD, end-stage renal disease; GN, glomerulonephritis; PKD, polycystic kidney disease; HD, hemodialysis; PD, peritoneal dialysis; HLA, human leukocyte antigen; ATG, anti-thymocyte globulin; CNI, calcineurin inhibitor; ANOVA, analysis of variance.

### 3.2 Donor plasma CIRBP as a risk factor for DGF after kidney transplantation

**Figure 2:**
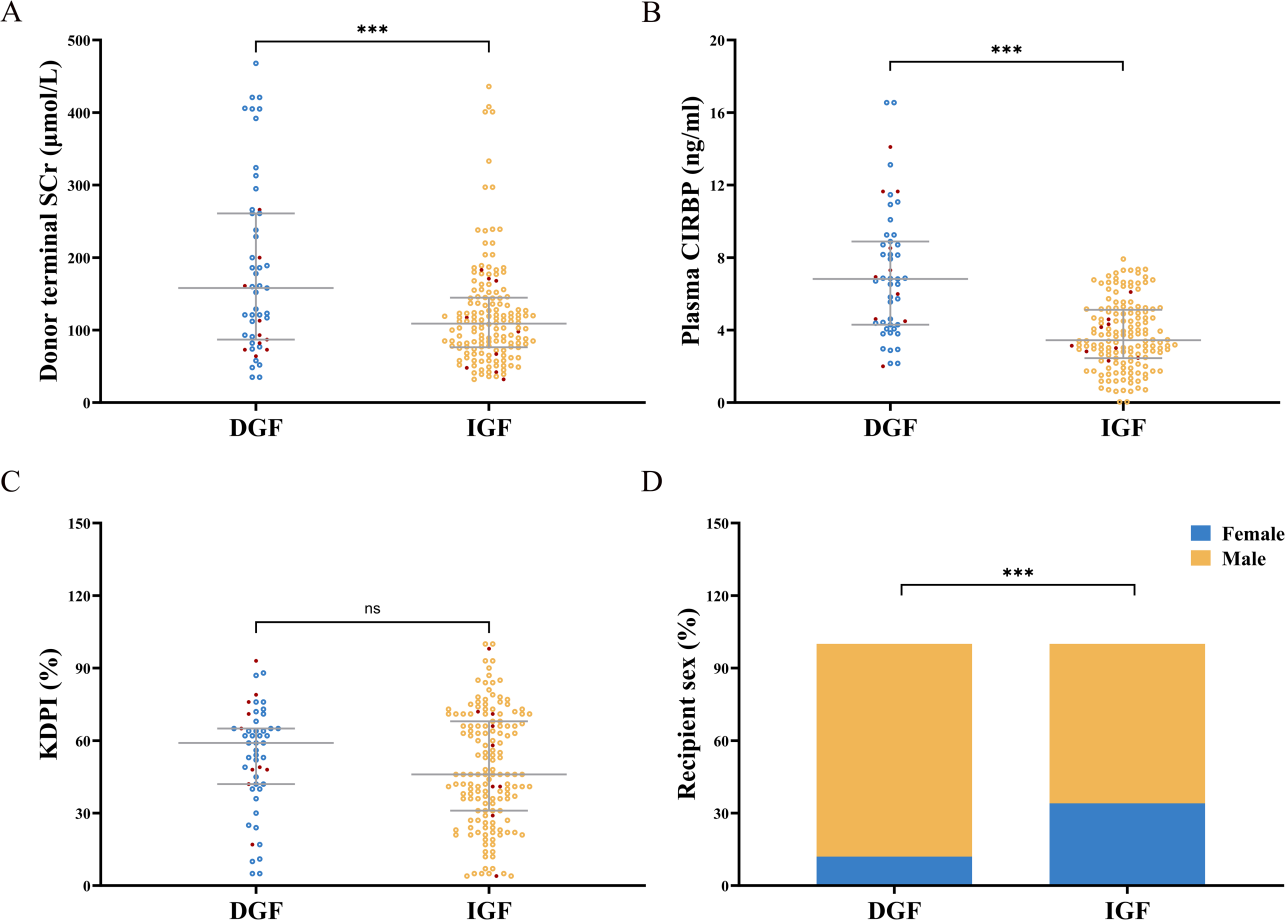
**(A)** Donor terminal serum creatinine (SCr) levels were significantly elevated in the DGF group compared to the IGF group. **(B)** Donor plasma CIRBP concentrations were markedly higher in the DGF group than in the IGF group. **(C)** There was no statistically significant difference in KDPI values between the DGF and IGF groups. **(D)** The proportion of female recipients in the DGF group was significantly lower compared to the IGF group. Red dots indicate lost graft kidneys. ns, no significance; ***, P<0.001.

DGF occurred in recipients paired with 51 (25%) donors following kidney transplantation. Testing of donor plasma samples revealed that the median CIRBP concentration in the DGF group was 6.82 ng/mL, which was 1.98 times higher than that in the IGF group (6.82 vs. 3.44; P<0.001). Recipients with CIRBP concentrations exceeding 5.484 ng/mL were four times more likely to develop DGF than those with concentrations below 5.484 ng/mL (53% vs. 12%; P<0.001). When CIRBP levels exceeded 7.92 ng/mL, the probability of developing DGF was 100%, whereas levels below 2 ng/mL corresponded to 0% probability of developing DGF (**Figure 2B**). Although the median terminal SCr concentration was notably higher in the DGF group than that in the IGF group, 19% of recipients developed DGF postoperatively even when the SCr concentration was < 100 μmol/L (**Figure 2A**). Additionally, the scatter distribution of CIRBP resembled that of SCr (**Figure 2**), and Spearman’s correlation demonstrated a significant relationship between the two factors (r=0.140, P=0.045). Furthermore, the proportion of female recipients was notably lower in the DGF group than that in the IGF group (12% vs. 34%; P=0.002) (**Figure 2D**), while no significant variation in KDPI was found between the two groups (**Figure 2C**). Graft loss occurred in 10 recipients in the DGF group, showing a higher rate, compared to the IGF group (19.6% vs. 5.8%; P=0.003).

To further clarify the role of CIRBP in DGF, a logistic regression analysis was conducted. Univariate analysis revealed that for every 1 μmol/L increase in donor terminal SCr, the risk of developing DGF increased by 0.7% (95% confidence interval [CI]: 1.004–1.010; P<0.001), for every 1 ng/mL rise in plasma CIRBP concentration, the risk increased by 73.3% (95% CI: 1.446–2.076; P<0.001), and female recipients had a 0.259-fold lower probability of developing DGF compared to males (95% CI: 0.104–0.646; P=0.004). Donor plasma CIRBP (OR=1.660; 95% CI: 1.376–2.004; P<0.001) and donor terminal SCr (OR=1.005; 95% CI: 1.001–1.009; P=0.025) were identified by multivariate analysis as independent risk factors for DGF development, while a protective factor was female sex of the recipient (OR=0.302; 95% CI: 0.103–0.885; P=0.029) (**Table 3**).

**Table 3:** Univariate and multivariate logistic regression analyses for the predictors of delayed graft function.

	Univariate			Multivariate		
	OR	95%CI	P	OR	95%CI	P
Donor age (y)	0.995	0.974 – 1.017	0.675			
Donor sex (women)	1.133	0.470 – 2.730	0.780			
Donor BMI (kg/m2)	1.009	0.910 – 1.118	0.870			
Donor cause of death (Cerebral hemorrhage)	0.350	0.280 – 1.418	0.324			
Donor terminal SCr (μmol/L)	1.007	1.004 – 1.010	0.001	1.005	1.001-1.009	0.025
Recipient age (y)	1.005	0.976 – 1.035	0.737			
Recipient sex (women)	0.259	0.104 – 0.646	0.004	0.302	0.103-0.885	0.029
Duration of dialysis before transplantation (mo)	1.002	0.989 – 1.016	0.725			
Level of HLA mismatch						
Level 2	0.771	0.236 – 2.517	0.667			
Level 3	0.407	0.122 – 1.353	0.143			
Plasma CIRBP (ng/ml)	1.733	1.446 – 2.076	0.001	1.660	1.376-2.004	0.001

Multivariate logistic regression analysis was performed with a backward selection procedure.

BMI, body mass index; SCr, serum creatinine; CI, confidence interval; OR, odds ratio; HLA, human leukocyte antigen; CIRBP, cold-inducible RNA binding protein.

### 3.3 Donor plasma CIRBP effectively predicts DGF

**Figure 3:**
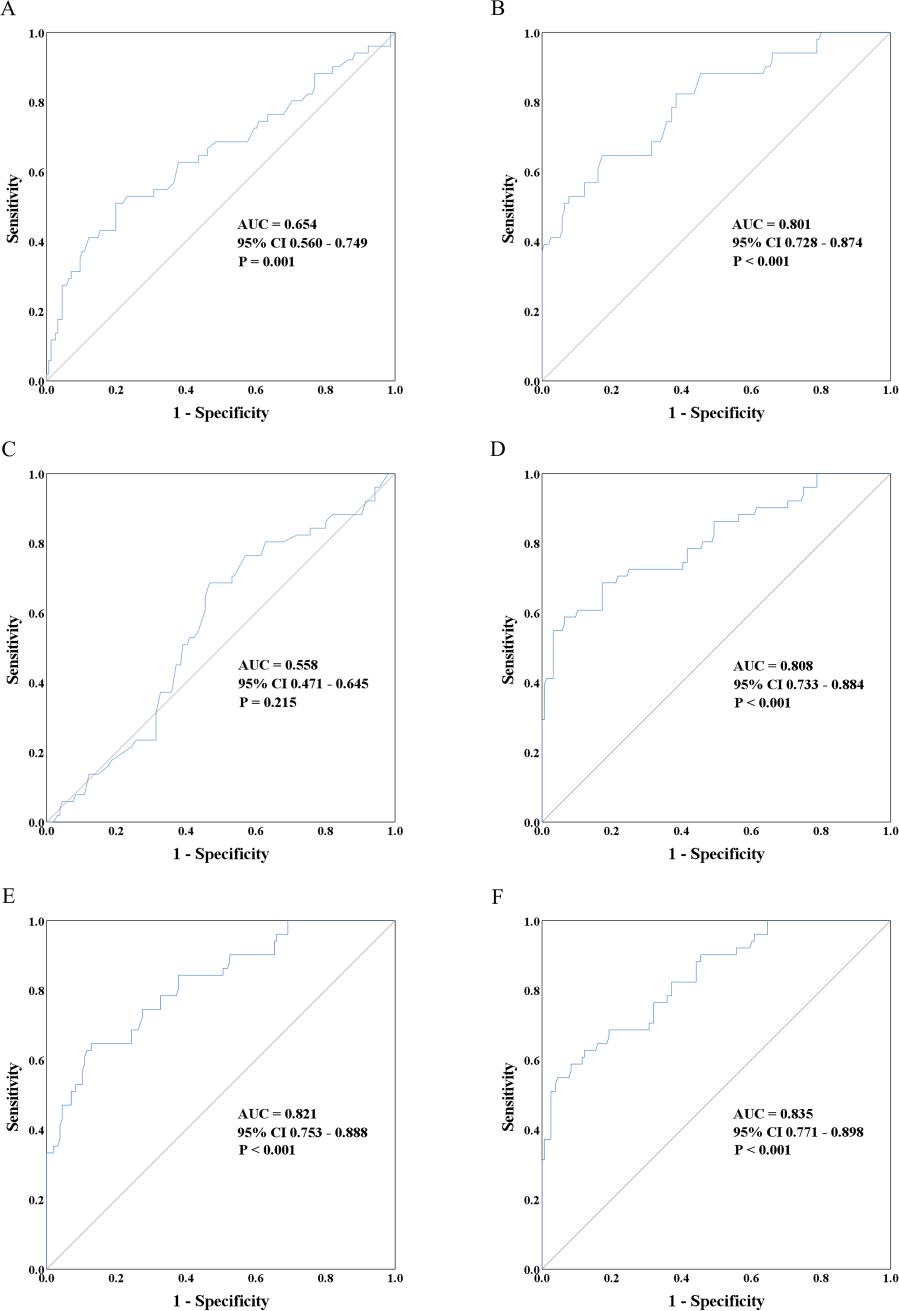
The receiver operating characteristic (ROC) curves depict the ability of donor characteristics to distinguish between immediate graft function (IGF) and delayed graft function (DGF). These curves include donor terminal serum creatinine **(A)**, donor plasma CIRBP **(B)**, kidney donor profile index (KDPI) **(C)**, a prediction model incorporating donor plasma CIRBP and terminal serum creatinine **(D)**, a prediction model incorporating donor plasma CIRBP and female recipient status **(E)**, and a prediction model combining donor plasma CIRBP, female recipient status, and terminal serum creatinine **(F)**.

The area under the ROC curve (ROC-AUC) of CIRBP was 0.801 (95% CI: 0.728–0.874; P<0.001), and the optimal cut-off value was 5.548 ng/mL, calculated using the Youden index, with a sensitivity of 65% and 83% specificity (**Figure 3B**). This performance was markedly superior to that of donor-terminal creatinine (ROC-AUC=0.654; 95% CI: 0.560–0.749; P=0.001) and KDPI (ROC-AUC=0.558; 95% CI: 0.471–0.645; P=0.215) (**Figures 3A, C**). Enhancing the predictive ability of CIRBP, ROC curve analysis was performed via logistic regression, separately fitting donor plasma CIRBP to donor terminal SCr levels and recipient sex. The best predictive model was represented by the formula: y=1.660×donor plasma CIRBP+3.312×male+1.005×donor terminal SCr-5.365 (ROC-AUC=0.835; 95% CI: 0.771–0.898; P<0.001). The optimal cutoff value was 0.308, having a sensitivity of 63% and 88% specificity (**Figure 3F**) and was superior to both the fitting model of plasma CIRBP and donor terminal SCr (ROC-AUC=0.808; 95% CI: 0.733–0.884; P<0.001) and the fitting model of plasma CIRBP and recipient sex (ROC-AUC 0.821; 95% CI: 0.753–0.888; P<0.001) (**Figures 3D, E**).

### 3.4 Correlation between donor plasma CIRBP and recipient creatinine at 6 months post-transplantation

**Figure 4:**
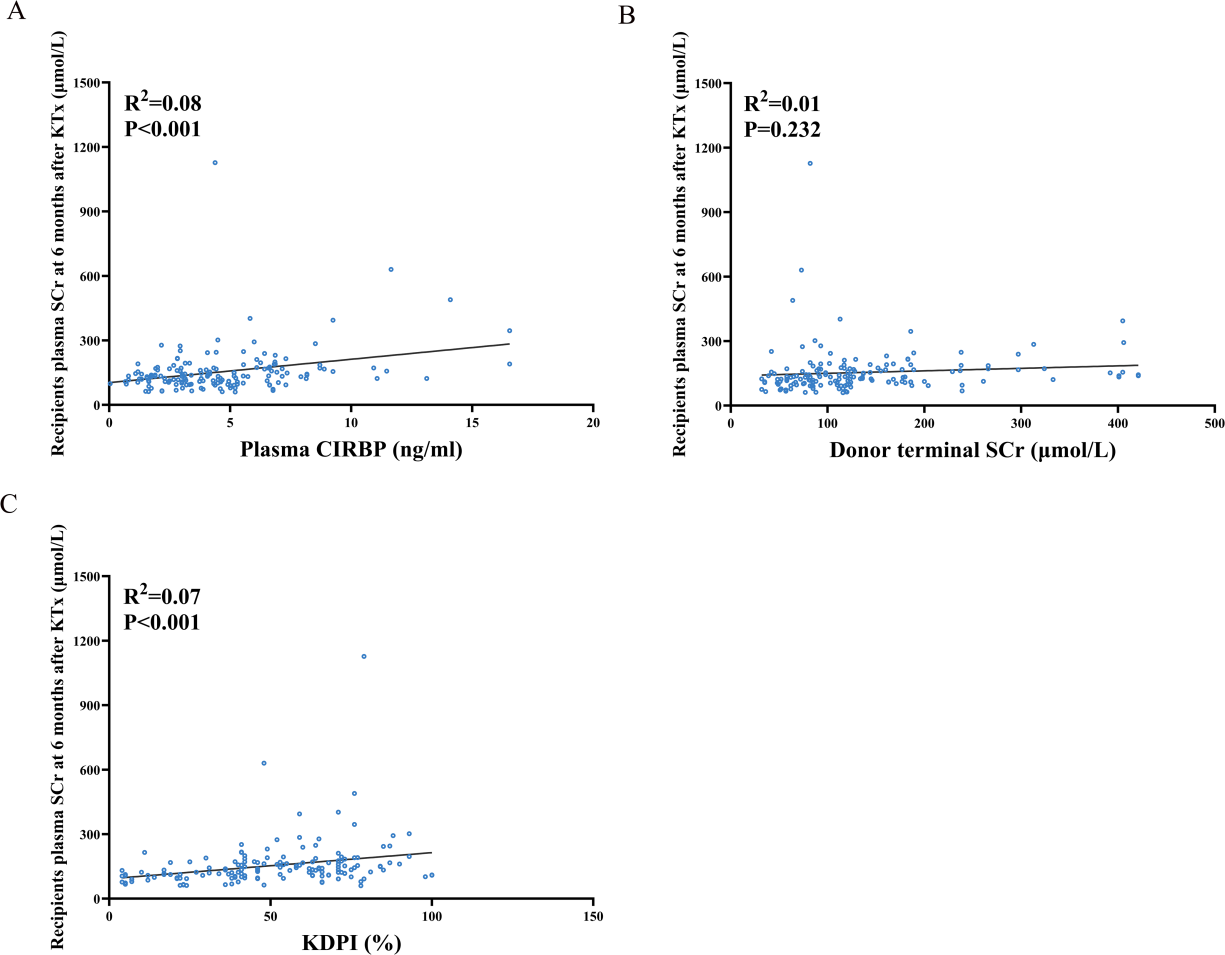
Spearman correlation analysis between recipient serum creatinine levels at 6 months post-transplantation and donor plasma donor CIRBP **(A)**, terminal serum creatinine **(B)**, and KDPI **(C)**.

At 6 months post-transplantation, recipient SCr concentration increased with increasing plasma CIRBP levels in donors (**Figure 4A**). Meanwhile, the SCr concentration at 6 months post-transplantation increased with the KDPI value (**Figure 4C**), consistent with previous findings, suggesting that the KDPI may help predict long-term donor kidney survival. However, no significant trend was noted for donor terminal SCr levels (**Figure 4B**).

Univariate linear regression analysis revealed significant linear correlations between SCr concentration at 6 months and donor plasma CIRBP (R²=0.08, P<0.001), KDPI (R²=0.07, P<0.001), female recipients (R²=0.06, P=0.002), donor age (R²=0.054, P=0.003), as well as donor cause of death being cerebral hemorrhage (R²=0.035, P=0.016) (**Table 4**). Ultimately, only donor plasma CIRBP (R²=0.08, P=0.003), KDPI (R²=0.137, P=0.002), and female recipient status (R²=0.174, P=0.008) were retained in the multiple linear regression analyses (**Table 4**). Conversely, donor terminal SCr levels were not found to be associated with recipient creatinine at 6 months post-transplantation.

**Table 4:** Univariate and multivariate linear regression analyses for the prediction of 6-month graft function.

	Univariate			Multivariate		
	β Coefficient	95%CI	P	β Coefficient	95%CI	P
Donor age (y)	1.749	0.605 to 2.894	0.003			
Donor sex (women)	21.726	-28.103 to 71.556	0.390			
Donor BMI (kg/m2)	0.978	-4.906 to 6.863	0.743			
Donor cause of death (Cerebral hemorrhage)	40.455	7.500 to 73.410	0.016			
Donor terminal SCr (μmol/L)	0.116	-0.075 to 0.306	0.232			
Recipient age (y)	-0.306	-1.939 to 1.327	0.712			
Recipient sex (women)	-56.821	-92.250 to -21.391	0.002	-46.068	-79.910 to -12.225	0.008
Duration of dialysis before transplantation (mo)	0.131	-0.545 to 0.806	0.703			
Level of HLA mismatch						
Level 2	-4.780	-69.109 to 59.548	0.884			
Level 3	-28.837	-92.460 to 34.787	0.372			
Plasma CIRBP (ng/ml)	10.874	5.273 to 16.474	0.001	8.358	2.874 to 13.843	0.003
KDPI	1.219	0.544 to 1.893	0.001	1.023	0.374 to 1.671	0.002

Multivariate linear regression analysis was performed using a stepwise variable selection procedure.

BMI, body mass index; SCr, serum creatinine; CI, confidence interval; HLA, human leukocyte antigen; CIRBP, cold-inducible RNA binding protein; KDPI, kidney donor profile index.

### 3.5 Donor plasma CIRBP and its association with transplanted kidney survival time

**Figure 5:**
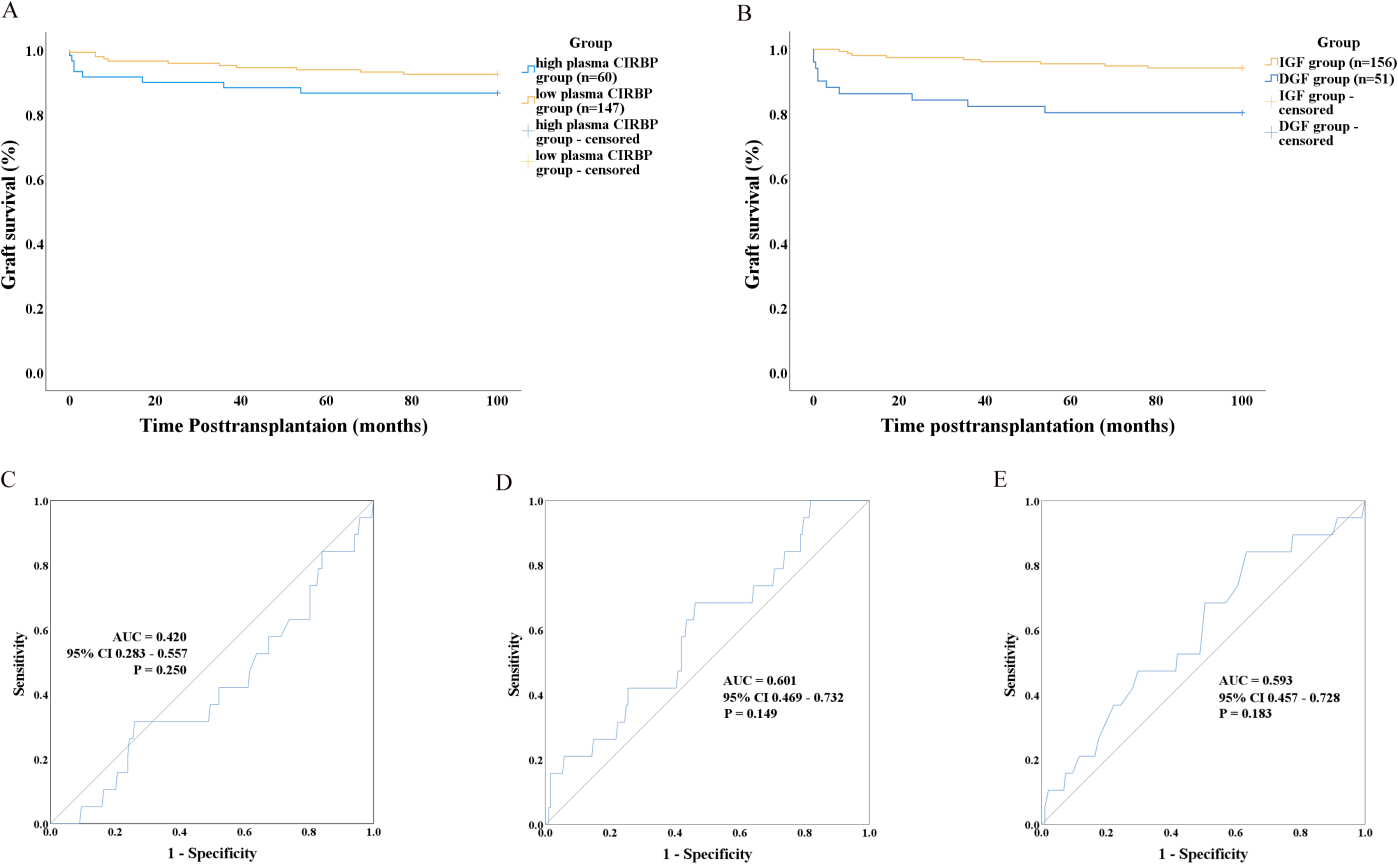
**(A)** Graft survival analysis using Kaplan–Meier curves based on donor plasma CIRBP levels (> 5.484 ng/ml). **(B)** Graft survival analysis using Kaplan–Meier curves according to whether recipients developed DGF. ROC curves showing the performance of donor characteristics in distinguishing between graft survival and graft loss, including donor terminal serum creatinine **(C)**, donor plasma CIRBP **(D)**, and KDPI **(E)**.

Recipients were categorized into high plasma CIRBP (≥5.484 ng/mL, n=60) and low plasma CIRBP (<5.484 ng/mL, n=147) groups based on the optimal cutoff value (**Figure 3B**). We hypothesized that donor kidneys with higher plasma CIRBP concentrations would exhibit significantly shorter survival times compared to those with lower concentrations. Although our survival analysis showed a trend of reduced survival time for donor kidneys with plasma CIRBP concentrations >5.484 ng/mL, further statistical analysis did not support this hypothesis (P=0.167; **Figure 5A**). However, when comparing graft survival times between the DGF and IGF groups, the Kaplan-Meier curves revealed that recipients who developed DGF had shorter long-term graft survival (P=0.002; **Figure 5B**), consistent with findings from related studies.

Univariate and multivariate analyses were conducted to identify the risk factors for kidney transplant loss. The results indicated that donor plasma CIRBP and donor terminal SCr were not significantly correlated with transplant loss (P=0.057 and P=0.231, respectively). Recipient female sex was the only significant factor correlated with reduced transplant loss risk (OR=0.125; 95% CI: 0.016–0.955; P=0.045) (**Table 5**). Subsequently, ROC curve analyses were conducted to evaluate the association between transplanted kidney survival and donor-terminal SCr (ROC-AUC=0.420; 95% CI: 0.283–0.557; P=0.250), plasma CIRBP (ROC-AUC=0.601; 95% CI: 0.469–0.732; P=0.149), and KDPI (ROC-AUC=0.593; 95% CI: 0.457–0.728; P=0.183) (**Figures 5C–E**). However, these analyses did not reveal any statistically significant differences.

**Table 5:** Univariate and multivariate logistic regression analyses for the predictors of graft loss.

	Univariate			Multivariate		
	OR	95%CI	P	OR	95%CI	P
Donor age (y)	1.023	0.986 – 1.061	0.233			
Donor sex (women)	1.662	0.512 – 5.397	0.398			
Donor BMI (kg/m2)	0.927	0.805 – 1.067	0.291			
Donor cause of death (Cerebral hemorrhage)	1.666	0.641 – 4.330	0.295			
Donor terminal SCr (μmol/L)	0.996	0.989 – 1.003	0.231			
Recipient age (y)	0.988	0.946 – 1.032	0.582			
Recipient sex (women)	0.125	0.016 – 0.955	0.045	0.125	0.016 – 0.955	0.045
Duration of dialysis before transplantation (mo)	1.012	0.996 – 1.028	0.146			
Level of HLA mismatch						
Level 2	0.750	0.146 – 3.847	0.730			
Level 3	0.438	0.081 – 2.352	0.335			
Plasma CIRBP (ng/ml)	1.149	0.996 – 1.325	0.057			

Multivariate logistic regression analysis was performed with a backward selection procedure.

BMI, body mass index; SCr, serum creatinine; CI, confidence interval; OR, odds ratio; HLA, human leukocyte antigen; CIRBP, cold-inducible RNA binding protein.

## 4 Discussion

To date, no study has established a correlation between CIRBP levels and kidney function after transplantation. In this study, CIRBP was identified for the first time as a risk factor for DGF development following kidney transplantation, demonstrating its potential to accurately predict DGF occurrence. Additionally, elevated donor plasma CIRBP concentrations were significantly associated with higher recipient SCr levels at 6 months post-transplant.

IRI is a major cause of AKI (30). During IRI, CIRBP is expressed intracellularly and released into the bloodstream via lysosomal and exosomal pathways, subsequently binding to TREM-1 receptors and exacerbating inflammation and kidney injury. Relative clinical studies demonstrated that elevated CIRBP concentrations correlated with an increased risk of AKI following cardiac macrovascular surgery (20, 21). Additionally, animal experiments by Cen et al. showed that IRI-induced kidney injury was significantly attenuated following CIRBP knockdown in mice (31). Therefore, we can hypothesize that elevated CIRBP concentrations in donor blood are associated with increased graft kidney injury severity and a higher DGF probability following kidney transplantation. In this study, ELISA was used to analyze donor plasma specimens. **Figure 2** demonstrates a significant correlation between plasma CIRBP and SCr concentrations. Higher plasma CIRBP concentrations were associated with elevated SCr levels, and the CIRBP concentration in donors from the DGF group was twice that of donors from the IGF group. Following further statistical analysis, we confirmed that CIRBP is an independent risk factor and an effective predictor of DGF after renal transplantation. Related animal experiments have shown that intravenous CIRBP injection induces AKI in mice, whereas the administration of a TREM-1 blocking agent significantly reduces IRI (32). In the future, blocking agents targeting CIRBP can be used in donors with elevated plasma CIRBP concentrations prior to renal transplantation, potentially reducing graft damage and the incidence of postoperative DGF.

In recent years, the exploration of predictors for DGF has continued; however, novel biomarkers generally face challenges, including complex detection methods, lack of uniform measurement standards, and the need for further cohort studies for validation (3), limiting their clinical application. Notably, researchers have also sought to combine and model DGF prediction using a variety of biomarkers that are easily acquired and detected but have limited predictive ability when used individually, achieving improved results. For instance, Rima et al. (2024) combined kidney injury molecule-1, interleukin-18, and clinical factors, reaching an ROC-AUC of 0.863 for DGF prediction (33). In our study, the ROC-AUC for DGF prediction using CIRBP alone was 0.801, and it shares the advantages of easy access to clinical specimens and convenient testing. Combining CIRBP with existing clinical models may enhance both the accuracy and clinical utility of DGF diagnosis.

Additionally, we observed that female recipients were significantly less likely to develop DGF than male recipients, suggesting a protective effect against DGF. Compared with men, women have higher levels of estrogen, which can increase autoantibody expression, inhibit Th1 cell-mediated responses, decrease the production of adhesion molecules as well as ROS in the vasculature, and elevate nitric oxide synthase expression, which are factors that play various roles in different diseases (34). Hence, we hypothesize that the inflammatory response in the vascular endothelium is attenuated by estrogen in women compared with men, leading to slower progression of chronic renal fibrosis in women, resulting in a reduced likelihood of DGF and extended survival of transplanted kidneys in female recipients. However, specific mechanisms underlying these effects in renal transplantation require further investigation.

Our findings revealed that elevated donor plasma CIRBP concentrations were associated with higher recipient SCr levels at 6 months post-kidney transplantation. Chronic graft dysfunction is a pressing issue in renal transplantation, primarily caused by chronic fibrosis and rejection following kidney injury (35). Bolourani et al. demonstrated that extracellular CIRBP triggers an inflammatory phenotype in pulmonary fibroblasts via a mechanism dependent on TLR4 (36). Therefore, it is reasonable to hypothesize that elevated plasma CIRBP concentrations are associated with more severe graft injury, leading to increased chronic renal fibrosis post-transplantation and resulting in higher recipient SCr levels at 6 months. However, this hypothesis requires further validation and targeting CIRBP may be a potential therapeutic strategy for chronic graft renal failure in future research. The survival time in the donor kidneys with elevated plasma CIRBP concentrations was not significantly different from that in the donor kidneys with low plasma CIRBP concentrations, although a decreasing trend was observed. During IRI, damaged cells release DAMPs that promote inflammation and activate and recruit T cells to the transplanted kidney via dendritic cells, exacerbating the injury and inducing rejection (37–39). CIRBP, a newly recognized DAMP molecule, shares similar properties, and its increased concentration should correlate with more severe kidney injury and a higher likelihood of transplant failure in the postoperative period. Possibly owing to the limited number of studies on recipients and the short follow-up period, female sex of recipients was the only observed protective factor that correlated with kidney graft loss in this study.

However, in our study, recipients who developed DGF after renal transplantation had longer graft survival compared to those without DGF, consistent with findings from related studies (40). DGF development significantly increases the incidence of posttransplant adverse events. In a 2021 study by Phillips et al. (41), of 4,714 kidney transplant recipients, 1,847 developed DGF, which gave rise to a 1.7-fold enhance in the risk of graft loss and a 1.8-fold increase in recipient death for those with DGF lasting more than 14 d. In a 2019 study, Kim et al. (42) reported a 2.4-fold increase in the 30-d readmission rate among 269 recipients who developed DGF. Similarly, Yousif et al. (43), in a 2022 study, reported a 1.6-fold increase in acute rejection among 578 renal transplant recipients who developed DGF. Therefore, prediction and prevention of DGF are crucial.

Unlike the extracellular CIRBP discussed herein, intracellular CIRBP plays a critical role in maintaining cellular stability by stabilizing RNA, preventing RNA degradation, and regulating RNA transcription (44). Related studies have shown that intracellular CIRBP in kidney cells can regulate the expression of hypoxia-inducible factor-1α, thereby reducing oxidative stress damage caused by IRI (45). Furthermore, a recent study demonstrated that CIRBP effectively attenuates hypothermic damage and inhibits ferroptosis in cardiac myocytes during heart transplantation (46). These findings suggest that, in the future, upregulating CIRBP expression in ECD donor kidneys while blocking the pathway of extracellular free CIRBP may reduce the risk of transplantation failure. This approach could potentially facilitate the re-expansion of the criteria for ECD donor kidneys and help address the critical shortage of donor kidneys.

Our study had certain limitations. It was a relatively small, retrospective, single-center study involving 207 cases, which restricted the range of factors available for analysis. Additionally, we were unable to obtain and analyze tissue specimens from zero-time biopsies of the transplanted kidneys. Plasma CIRBP levels in donors can be influenced by inflammation or trauma to the central nervous system (47), and as a result, are expected to vary with the duration of brain death in brain-dead donors. However, our study did not account for the important factor of donor brain death duration. In the future, it will be essential to collect additional zero puncture specimens from donor kidneys for pathological testing, record the duration of brain death and related confounders for analysis and conduct a multicenter, large-sample study to further validate our findings.

In conclusion, our preliminary study identified a correlation between CIRBP and transplant renal function, establishing donor plasma CIRBP as an independent risk factor for DGF after renal transplantation. Therefore, donor plasma CIRBP levels are expected to become a new index for evaluating transplanted kidney function and antagonizing CIRBP may represent a novel therapeutic approach to reducing the risk of kidney transplant failure, particularly in ECD donor kidney transplantation.

## Data Availability

The raw data supporting the conclusions of this article will be made available by the authors, without undue reservation.
